# Prevalence of Free Flap Failure in Patients Undergoing Reconstruction for Medication-Related Osteonecrosis of the Jaw: A Systematic Review and Meta-Analysis

**DOI:** 10.3390/clinpract15080151

**Published:** 2025-08-14

**Authors:** Evangelos Kostares, Georgia Kostare, Michael Kostares, Fani Pitsigavdaki, Athanassios Kyrgidis, Christos Perisanidis, Maria Kantzanou

**Affiliations:** 1 Department of Microbiology, Medical School, National and Kapodistrian University of Athens, 115 27 Athens, Greece; gkostare@med.uoa.gr (G.K.);; 2 Department of Anatomy, Medical School, National and Kapodistrian University of Athens, 115 27 Athens, Greece; michaliskost@med.uoa.gr; 3 Department of Oral and Maxillofacial Surgery, Dental School, National and Kapodistrian University of Athens, 115 27 Athens, Greece; 4 Department of Oral and Maxillofacial Surgery, General Hospital of Thessaloniki “George Papanikolaou”, Aristotle University of Thessaloniki, 57010 Thessaloniki, Greece

**Keywords:** free tissue flaps, osteonecrosis, jaw, prevalence, treatment failure, meta-analysis

## Abstract

**Background/Objectives**: Medication-related osteonecrosis of the jaw (MRONJ) is a serious complication in patients treated with antiresorptive or antiangiogenic agents, particularly those with cancer-related comorbidities. This systematic review and meta-analysis aimed to estimate the prevalence of free flap failure in patients undergoing microvascular reconstruction for MRONJ. **Methods**: A comprehensive literature search was conducted across Medline/PubMed, Scopus, and Web of Science up to 30 January 2025. Inclusion criteria were observational studies involving MRONJ patients treated with free flap reconstruction. Risk of bias was assessed using the Newcastle–Ottawa Scale. The pooled prevalence of free flap failure was calculated using a random-effects model with Freeman–Tukey double arcsine transformation. **Results**: Twelve studies were included in the quantitative analysis. The fibula free flap was the most frequently used flap. The pooled prevalence of free flap failure was 0.1% (95% CI: 0–2.3%), with no significant associations observed in meta-regression analyses for publication year, patient age, or sex. All included studies were of moderate methodological quality. **Conclusions**: These findings suggest that free flap reconstruction is a reliable and effective surgical option for managing advanced MRONJ in well-resourced and specialized healthcare settings; however, limitations such as small sample sizes and heterogeneity in protocols must be considered. Further high-quality, multicenter studies are needed to evaluate long-term outcomes and refine perioperative management strategies.

## 1. Introduction

Medication-related osteonecrosis of the jaw (MRONJ) is defined as a progressive destruction and necrosis of the jawbone in patients treated with antiresorptive or antiangiogenic agents, without prior radiation therapy or metastatic disease to the jaws. It remains a significant complication associated with these medications [1,2]. A recent meta-analysis by Zhang Y. et al. [3], which included 23 studies involving 32,815 patients, estimated the prevalence of MRONJ in patients treated with denosumab or bisphosphonates to be 2.08% (95% confidence interval (CI): 1.37–2.91%). The authors also found that denosumab was associated with a significantly higher risk of MRONJ compared to zoledronic acid (risk ratio (RR) = 1.49; 95% CI: 0.99–2.25). Similar a meta-analysis conducted by Srivastava A., et al. [4] found that the prevalence of MRONJ was significantly higher in patients treated with sequential ARD therapies, such as pamidronate–zoledronate (19%, 95% CI:10–27%) and bisphosphonate–denosumab (13%, 95% CI:3–22%), compared to single-agent therapies like denosumab alone (4%, 95% CI 3–5%) or bisphosphonates alone (5%, 95% CI: 0–9%). An observational study by D’Agostino S., et al. [5] found that the majority of MRONJ patients presented with poor oral hygiene, suggesting it could be a contributory factor in disease progression; however, no significant statistical correlation was observed. Among local risk factors, it is also worth mentioning that endodontic treatment failure may predispose to MRONJ development; a study by Tempesta A., et al. [6] demonstrated that in the absence of conventional triggers such as extractions or ill-fitting dentures, failed root canal treatments were the only identifiable local factor in a series of oncologic patients who developed MRONJ.

Over the past two decades, efforts to standardize its definition, diagnosis, and management have led to the development of national guidelines by various professional societies. The American Association of Oral and Maxillofacial Surgeons (AAOMS) has periodically updated its position papers, most recently in 2022, emphasizing a clinical staging system that categorizes MRONJ into four stages: “at-risk,” Stage 0 (non-exposed variant), and Stages 1 to 3, based primarily on clinical signs such as bone exposure, symptoms, and the presence of infection or anatomical complications. In contrast, the 2024 Italian position paper jointly developed by the Italian Society of Oral Pathology & Medicine (SIPMO) and the Italian Society of Maxillofacial Surgery (SICMF) introduces a more comprehensive diagnostic and staging framework, integrating clinical signs with radiological findings—particularly CT-based evidence of bone marrow sclerosis and cortical erosion. The Italian classification system aims to enhance diagnostic accuracy, especially for early or non-exposed forms of MRONJ, and supports imaging as a fundamental tool for disease staging and treatment planning. Furthermore, the SIPMO–SICMF panel offers a more granular stratification of patient risk categories and places greater emphasis on individualized prevention strategies, including therapeutic drug holidays. This article aims to compare the key differences and commonalities between these two authoritative guidelines, with a particular focus on staging criteria and their implications for clinical decision-making [1,2].

According to AAOMS [1], management of MRONJ is guided by disease staging, with treatment goals including control of infection, pain reduction, and prevention of lesion progression. Conservative approaches, such as antimicrobial mouth rinses, systemic antibiotics, and pain control, are recommended for early stages, especially Stage 1, where patients are asymptomatic. Surgical intervention, including debridement or resection, may be warranted in advanced cases (Stages 2 and 3) presenting with infection, fistulae, or extensive necrosis. In severe Stage 3 cases with complications such as pathologic fractures or oroantral communications, resection followed by reconstruction with vascularized free tissue transfer (free flap reconstruction) may be indicated. Multidisciplinary care, continued medical therapy when appropriate, and patient education are emphasized to balance MRONJ management with ongoing antiresorptive or antiangiogenic treatments. Preventive dental assessments and minimizing dentoalveolar surgery in high-risk patients are key strategies to reduce MRONJ incidence [1].

While previous systematic reviews have explored free flap outcomes in the context of osteoradionecrosis, to our knowledge, no prior study has systematically examined the prevalence of free flap failure specifically in patients undergoing reconstruction for MRONJ. MRONJ is a distinct clinical condition, differing in its pathophysiology, treatment considerations, and patient population. As such, it warrants a focused analysis separate from osteoradionecrosis. This article contributes to the field by presenting the first systematic review and meta-analysis focused on calculating the prevalence of free flap failure in MRONJ patients, synthesizing the available evidence to address a critical gap in the literature regarding the outcomes of advanced reconstructive procedures in this population.

## 2. Materials and Methods

### 2.1. Search Strategy

A systematic literature search was independently conducted by two reviewers (E.K., G.K.) in Medline/PubMed Central (PMC) (via PubMed), Scopus, and Web of Science, covering publications up to 30 January 2025. The study adhered to the structure and reporting standards outlined in the Preferred Reporting Items for Systematic Reviews and Meta-Analyses (PRISMA) guidelines. The PRISMA checklist is available in the Supplementary Materials (Supplementary Table S1). The search algorithm included keywords such as: bisphosphonate, denosumab, medicat *, antiresorptive, osteonecrosis, MRONJ, free flap, free tissue, vascul *, fibula, radial forearm, iliac crest, scapula, mandib *, maxill *, jaw. The complete search strategy for each database is provided in the Supplementary Materials (Supplementary Table S2).

Two independent researchers (E.K., G.K.) thoroughly examined the publications identified in the initial search. First, duplicates were removed. The titles and abstracts of the remaining articles were then screened, and publications that did not meet the predetermined inclusion criteria were excluded. The full texts of the remaining articles were obtained and carefully assessed. During this process, the reference lists of eligible articles were reviewed to identify any potentially missed studies. Zotero version 7.0.16 (Corporation for Digital Scholarship, Vienna, Virginia, USA) reference management software was used to support this process. Any discrepancies were resolved through team consensus.

### 2.2. Criteria for Study Selection and Data Extraction

In our systematic review and proportional meta-analysis, we developed our inclusion criteria based on the Population, Exposure, Comparison, Outcomes, and Study Types (PECOS) framework, as outlined in Table 1.

To summarize, this review included observational studies focusing on patients with MRONJ who underwent free flap reconstruction and reported free flap failure as an outcome. Studies involving osteoradionecrosis, pediatric patients, non-free flap reconstructions, or those with limited methodological rigor were excluded. A detailed description of all inclusion and exclusion criteria is provided in Table 1.

### 2.3. Data Extraction

The following information was collected from each study (M.K., F.P.): primary author’s name, year of publication, study design, continent and country of origin, study duration, total number of patients with MRONJ, total number of free flaps used, gender distribution (proportion of males), mean age, proportion of fibula flaps used, number of free flap failures, number of MRONJ recurrences, indication for antiresorptive or antiangiogenic medication and the type of medication used.

### 2.4. Quality Assessment

Each study was thoroughly assessed by two independent researchers using the Quality Assessment Tools developed through a collaboration between the Universities of Newcastle, Australia, and Ottawa, Canada. The modified Newcastle–Ottawa Scale (NOS) was applied for cross-sectional studies, while the original NOS was used for cohort studies. The objective of this evaluation was to identify any potential methodological or survey-related issues that might compromise the internal validity of the research. Studies were scored based on three main criteria: the selection of study groups, the comparability of those groups, and the ascertainment of exposure or outcome for case–control or cohort studies (or for cross-sectional studies using the modified approach). A “star system” was employed for this assessment. Studies that received scores between 7 and 9 were considered to be at low risk of bias (high quality), those scoring between 4 and 6 were rated as moderate quality, and those with scores from 0 to 3 were regarded as having a high risk of bias (low quality) [7].

### 2.5. Statistical Analysis

Statistical analysis was conducted using RStudio software (RStudio, PBC, USA; version 2022.12.0+353). As planned, we performed a meta-analysis using the metafor package [8] to estimate pooled prevalence and 95% confidence intervals (CI), applying the DerSimonian and Laird random-effects model. The Freeman–Tukey double arcsine transformation was used to stabilize variances [9].

Heterogeneity among studies was assessed through visual inspection of the forest plot, Cochran’s Q statistic (with *p* value), and the Higgins I^2^ statistic with 95% CI. The I^2^ values were interpreted using the following thresholds: 0–40% (not important), 30–60% (moderate), 50–90% (substantial), and 75–100% (considerable heterogeneity).

Meta-regression analysis was performed when at least ten studies were available for a given continuous or categorical variable [10]. Sensitivity analysis was conducted using a leave-one-out approach, in which each study was sequentially excluded to examine its impact on the overall estimate. Unless otherwise specified, statistical significance was set at *p* = 0.05 (two-tailed).

Publication bias was assessed qualitatively. Although methods such as Egger’s test, Begg’s test, and funnel plots are often used in comparative data, they were not applied here due to the lack of consensus on defining positive results in meta-analyses of proportions [11,12].

## 3. Results

### 3.1. Results and Characteristics of the Included Studies

**Figure 1:**
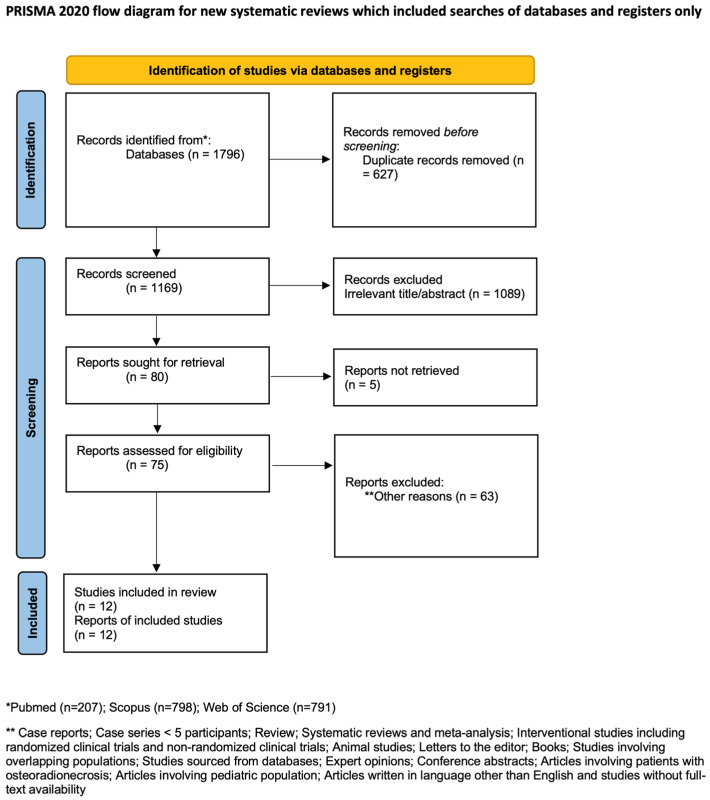
PRISMA flowchart (Source: Page MJ, et al. BMJ 2021;372:n71. doi: 10.1136/bmj.n71. [25]).

Of the 1796 articles identified in the databases, 12 studies were ultimately included in the quantitative analysis [13,14,15,16,17,18,19,20,21,22,23,24] after removing duplicates, screening titles and abstracts, and assessing full texts against our inclusion criteria. The PRISMA flow chart illustrating the selection process is presented in Figure 1. Table 2 summarizes their descriptive characteristics. The included studies were published between 2009 and 2025 and conducted between 2004 and 2024. Ten studies were cohort designs [13,14,16,17,18,19,20,21,23,24], while two were case series [15,22]. A total of 171 patients with MRONJ were included, all of whom underwent reconstruction with free flaps. Gender data were available in all 12 studies [13,14,15,16,17,18,19,20,21,22,23,24], with males comprising 32.1% of patients. Mean age was reported in 11 studies [13,14,15,16,17,18,20,21,22,23,24], with a median value of 64.7 years.

In terms of surgical technique, the fibula free flap was used exclusively in 7 of the 12 studies [13,14,16,17,21,23,24] and was the most commonly employed flap overall, representing 83.6% (143/171) of all reconstructions. The remaining 28 patients received other types of free flaps. Recurrence of MRONJ was reported in just three studies and affected a total of eight patients. The studies spanned three continents—Europe (6 studies) [13,17,19,20,21,24], North America (4 studies) [15,18,22,23], and Asia (2 studies) [14,16] and were conducted in Germany, Italy, Switzerland, the USA, and China. The most common clinical indications for antiresorptive or antiangiogenic medication use among patients were breast cancer (n = 45), multiple myeloma (n = 30), and prostate cancer (n = 18); however, additional conditions such as lung cancer, osteoporosis, malignant plasmacytoma, lymphoma, renal cell carcinoma, and other unspecified malignancies were also reported. Nine studies specifically mentioned the medications used. In total, bisphosphonates were implicated in 111 patients, either as a single agent or in combination with denosumab. Denosumab was specifically reported in three studies and was used in 6 patients. All included studies were assessed as being of moderate methodological quality, based on the criteria applied (Supplementary Table S3).

### 3.2. Prevalence of Free Flap Failure in MRONJ Patients Undergoing Reconstruction

**Figure 2:**
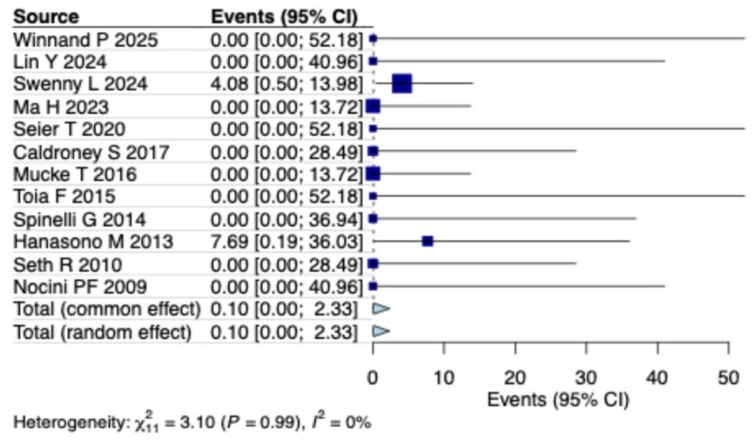
Forest plot illustrating the prevalence of free flap failure in patients with MRONJ [13,14,15,16,17,18,19,20,21,22,23,24].

A random-effects model analysis estimated the prevalence of free flap failure in MRONJ patients undergoing reconstruction at 0.1% (95% CI: 0–2.3%), with not important heterogeneity between studies (I^2^ = 0%) (Figure 2). Leave-one-out analysis showed consistent results, with no single study markedly affecting the pooled estimate (Supplementary Figure S1).

### 3.3. Meta-Regression Analysis

To investigate potential sources of heterogeneity, a meta-regression analysis was conducted evaluating the impact of continuous variables on the free flap failure in MRONJ patients undergoing reconstruction. Specifically, the year of publication, the proportion of male patients, and the mean age were examined. None of these variables demonstrated a statistically significant association with free flap failure prevalence (Supplementary Table S4).

## 4. Discussion

This systematic review and meta-analysis assessed the prevalence of free flap failure in patients undergoing reconstruction for MRONJ, a rare but severe complication of antiresorptive and antiangiogenic therapy. Our analysis, encompassing 171 patients across 12 studies, revealed an overall low failure rate of free flap reconstructions, highlighting the reliability of this approach, even in complex cases. The fibula free flap emerged as the most frequently utilized and successful reconstructive option, employed in over 80% of cases. These findings align with existing literature that supports the fibula flap’s versatility, robust vascularity, and suitability for extensive mandibular defects. Notably, recurrence of MRONJ was infrequently reported and limited to a few studies, suggesting that vascularized bone reconstruction may play a protective role against disease recurrence.

In our recently published systematic review and meta-analysis evaluating outcomes of free flap reconstruction in mandibular osteoradionecrosis, we analyzed data from 46 studies encompassing 1344 free flaps. The pooled prevalence of total free flap failure was relatively low at 3.1% (95% CI: 1.3–5.4%), despite the complexity of cases and prior radiation exposure. This supports the reliability of free tissue transfer in challenging reconstructive scenarios. Notably, no influential outliers were identified, and meta-regression did not reveal significant moderators, suggesting a consistent benefit across diverse clinical contexts [26].

In addition, Farsi S., et al. [27] (2025) conducted a systematic review and meta-analysis evaluating the outcomes of free flap reconstruction in patients with advanced mandibular osteoradionecrosis. Their analysis included 19 studies encompassing 424 free flap procedures and revealed a pooled prevalence of postoperative complications, excluding flap failures, such as surgical site infections, fistula formation, skin paddle necrosis, nonunion, and plate exposure, at 22.5% (95% CI: 16.5–28.6%). Among the different donor sites, radial forearm flaps had the lowest complication rate at 13.4% (95% CI: 2.6–29.7%), while scapular flaps showed the highest at 34.9% (95% CI: 3.9–66.0%). Importantly, most complications were managed successfully. The pooled data also demonstrated favorable functional outcomes, with high rates of restored alimentation and speech. Recurrence of osteoradionecrosis was observed in only 2.1% of cases, supporting the effectiveness of vascularized bone reconstruction in both disease control and functional rehabilitation.

While successful microvascular reconstruction provides a stable and vascularized osseous foundation, the ultimate goal extends beyond structural repair to include full functional rehabilitation of the patient. Zhang X., et al. [28] (2023) conducted a systematic review and meta-analysis assessing outcomes of dental implant rehabilitation in patients who underwent mandibular reconstruction. Their analysis of 32 studies involving 3515 implants revealed a high overall implant survival rate of 92.4%, with a pooled 3-year survival of 95.2% and a 5-year survival of 85.4%. Notably, the timing of implant placement, immediate versus delayed, did not significantly affect long-term outcomes. These findings highlight the viability of implant-supported prosthetic rehabilitation in reconstructed jaws and underscore the importance of integrating dental restoration into the overall treatment plan. Therefore, modern mandibular reconstruction should aim not only to reestablish mandibular continuity but also to restore masticatory function, aesthetics, and patient quality of life through implant-based rehabilitation.

The management of MRONJ remains individualized, as no definitive guidelines exist, with both conservative and surgical approaches employed based on disease stage and patient condition. Conservative treatments include antibiotics, analgesics, antiseptic rinses, and adjuvant therapies such as ultrasound and hyperbaric oxygen; however, the latter lacks consistent evidence of benefit [28]. Surgical interventions range from sequestrectomy to extensive resection and reconstruction in advanced cases. Controversy persists over drug holidays due to the long half-life of bisphosphonates [29,30]. Emerging therapies like piezosurgery and photobiomodulation offer promising minimally invasive alternatives, while the use of autologous platelet concentrates (e.g., L-PRF, PRF) has shown favorable outcomes in enhancing tissue healing and reducing postoperative complications [29,30].

### Study’s Limitations

Twelve studies were finally included. The overall sample size was relatively small (171 patients), with six studies enrolling fewer than 10 patients each. Additionally, the procedures were performed in specific centers and among selected populations. Different types of free flaps were used across studies, and peri- and postoperative protocols (e.g., antibiotics, follow-up schedules) varied. There were also differences in the indications for medication therapy and the medications used by patients. The studies were conducted across various geographic regions and time periods. Additionally, the lack of continuous and categorical variables prevented us from performing more comprehensive meta-regression and subgroup analyses. Another significant limitation was the exclusion of non-English language studies and those without full-text availability. Our search strategy involved the use of filters, which, while intended to improve the specificity of the search, may have inadvertently excluded some relevant studies. We attempted to mitigate this potential limitation through a careful manual search of the reference lists of all included articles to identify any publications that may have been missed. Moreover, the meta-analysis was not registered in PROSPERO, which may introduce bias, particularly selective reporting bias and researcher bias. Given these considerations, it is important to acknowledge that, as with almost any meta-analysis [31,32], our study is subject to potential biases, including selection bias, publication bias, language bias, and search bias, all of which may affect the validity and generalizability of our findings.

## 5. Conclusions

This systematic review and meta-analysis showed that free flap reconstruction for MRONJ is associated with a low prevalence of total flap failure. Several studies reported a lower recurrence rate of MRONJ when vascularized bone flaps were used. Despite variations in flap selection and perioperative protocols, the included studies consistently reported favorable reconstructive outcomes. However, small sample sizes and methodological heterogeneity limit the strength of these findings. Further large-scale, prospective studies are warranted.

## Figures and Tables

**Table 1:** Eligibility criteria.

Eligibility Criteria	Inclusion Criteria	Exclusion Criteria
Population (P)	Patients with MRONJ underwent free flap reconstruction	Patients with osteoradionecrosis, pediatric population
Exposure (E)	Free flap reconstruction	Other types of flaps (e.g., regional, etc.)
Comparison (C)	As our aim was to quantify prevalence, direct comparisons were not applicable	-
Outcomes (O)	Free flap failure	Other complications (infection, etc.)
Study Types (S)	Observational studies including cohort, case–control, and cross-sectional studies	Case reports, case series with <5 participants, review articles, systematic reviews, meta-analysis, interventional studies including randomized clinical trials and non-randomized clinical trials, comparative studies, animal studies, letters to the editor, books, studies involving overlapping populations, studies sourced from databases, expert opinions, conference abstracts, articles written in language other than English, and studies without full-text availability

**Table 2:** Detailed characteristics of the studies that were included in the evaluation.

First Author	Year of Publication	Study Design	Continent	Country	Study Period	Total Number of Patients	Free Flap Used	Proportion of Males (%)	Mean Age (Years)	Fibula Flaps Used (%)	Free Flap Failures	MRONJ Recurrence	Indication for Medication Use	Type of Medication	Quality Assessment
Winnand P [13]	2025	Cohort	Europe	Germany	2012–2023	5	5	0	68.6	100	0	NA	NA	NA	Moderate
Lin Y [14]	2024	Cohort	Asia	China	2023–2024	7	7	42.9	61.3	100	0	0	BC:2 MM:2 LC:3	B: 7 D: 3	Moderate
Swenny L [15]	2024	Case series	North America	USA	2010–2022	49	49	37	66	69.4	2	6	BC:20 MM:12 PC:9 MP:2 L:1 RCC:1 O: 4	NA	Moderate
Μa H [16]	2023	Cohort	Asia	China	1990–2022	25	25	36	63	100	0	NA	NA	B: 24 D: 1	Moderate
Seier T [17]	2020	Cohort	Europe	Switzerland	2015–2018	5	5	60	67	100	0	NA	NA	NA	Moderate
Caldroney S [18]	2017	Cohort	North America	USA	2010–2015	11	11	36	65.8	63.6	0	0	BC: 4 MM: 3 Os: 3 PC:1	B: 9 B plus D: 2	Moderate
Mücke T [19]	2016	Cohort	Europe	Germany	2007–2014	25	25	NA	NA	36	0	1	NA	B: 25	Moderate
Toia F [20]	2015	Cohort	Europe	Italy	2008–2013	5	5	40	63	0	0	0	BC:3 LC:1 PC:1	B: 5	Moderate
Spinelli G [21]	2014	Cohort	Europe	Italy	2004–2008	8	8	37.5	64.7	100	0	0	BC:3 PC:1 MM:4	B: 8	Moderate
Hanasono M [22]	2013	Case series	North America	USA	2007–2012	13	13	30.8	66.6	84.6	1	0	BC:3 PC:2 MM:5 Os:3	B: 13	Moderate
Seth R [23]	2010	Cohort	North America	USA	2006–2009	11	11	18.1	61.3	100	0	0	BC: 5 PC:2 MM:2 Os:2	B:11	Moderate
Nocini PF [24]	2009	Cohort	Europe	Italy	2004–2007	7	7	14.3	61	100	0	1	BC:5 MM:2 Os:2	B: 7	Moderate

NA: not applicable, BC: Breast cancer, MM: Multiple myeloma, PC: Prostate cancer, LC: Lung cancer, MP: Malignant plasmacytoma, L: Lymphoma, RCC: Renal cell carcinoma, Os: Osteoporosis, O: Other, B: Bisphosphonate, D: Denosumab.

## Data Availability

The original contributions presented in this study are included in the article/Supplementary Materials. Further inquiries can be directed to the corresponding author.

## Supplementary Materials

The Supplementary Materials for this study can be found at https://doi.org/10.5281/zenodo.15730890.
